# Supplementary material for: Dynamic full-field optical coherence tomography allows live imaging of retinal pigment epithelium stress model
Source: Commun Biol. 2022 Jun 10;5:575. doi: 10.1038/s42003-022-03479-6 (PMC9187748; doi:10.1038/s42003-022-03479-6)
Supplement: Supplementary file 1 — Supplementary information [file 42003_2022_3479_MOESM1_ESM.pdf]

Supplementary information

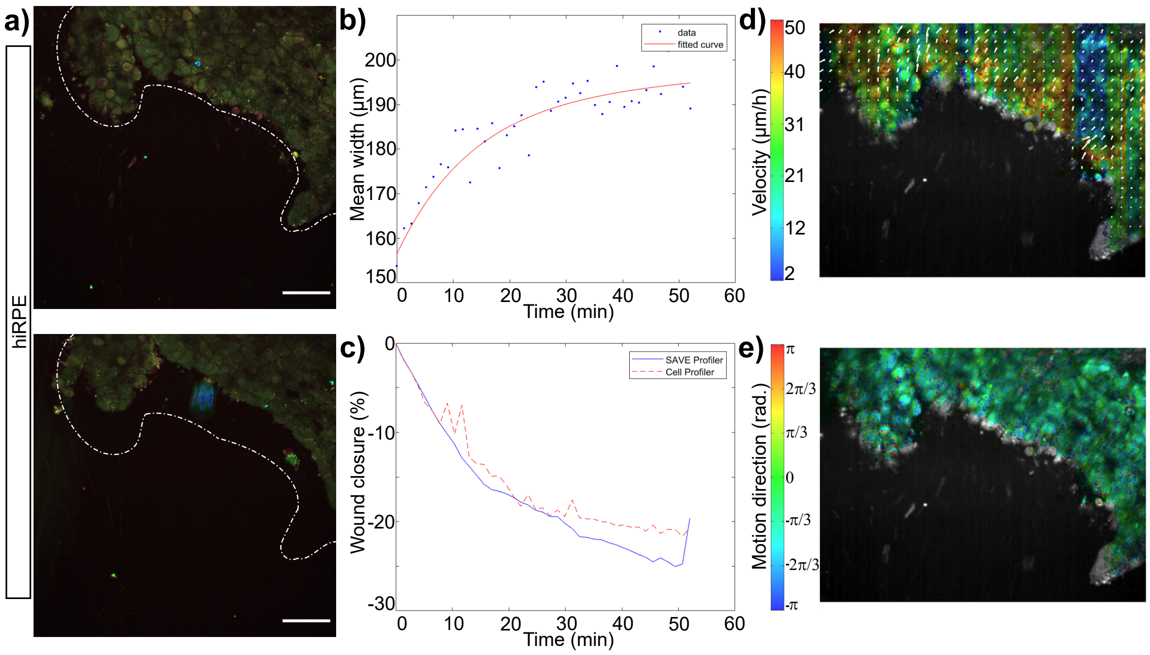

Supplementary Figure 1: **Results on expanding scratch assay on hiRPE cell culture. a)** Beginning and end of the imaging of an expanding scratch assay on hiRPE cell culture. The white dotted line represents the border of the wound at the beginning of the acquisition. **b)** Evolution of scratch width over the acquisition, calculated with our program. **c)** Wound closure calculated with both SAVE Profiler and the Cell Profiler software. **d) & e)** Results of the optic flow calculations showing both the velocity and the direction separately. (Scalebar: 50 μm)

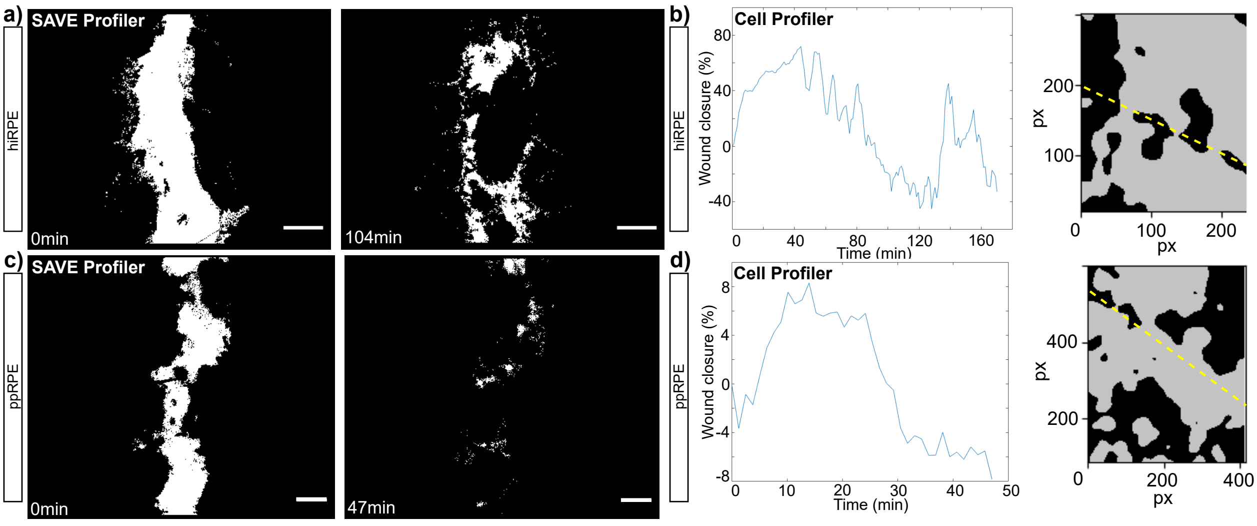

Supplementary Figure 2: **Comparison between SAVE Profiler and Cell Profiler on small wounds (closing). a) & b)** Comparison on hiRPE sample. **a)** shows the results of SAVE Profiler segmentation on the hiRPE closing wound shown in Fig.1, calculated over the zone indicated by the dotted white square on Fig.1d). Note that SAVE profiler rotates and crops the native D-FFOCT image to place the scratch vertically for the segmentation step, in contrast to Cell Profiler. **b)** Wound closure and segmentation calculated with Cell Profiler (scratch shown in dashed yellow line, in native diagonal orientation) on the same zone from Fig 1d), showing the poorer segmentation of Cell Profiler. **c) & d)** Comparison on a ppRPE sample. **c)** SAVE Profiler segmentation on the ppRPE closing wound shown in the dotted white square of Fig. 1h). **d)** Wound closure and segmentation calculated with Cell Profiler (scratch shown in dashed yellow line) on the same sample, again showing the superior performance of SAVE Profiler. (Scalebar: 10 μm)

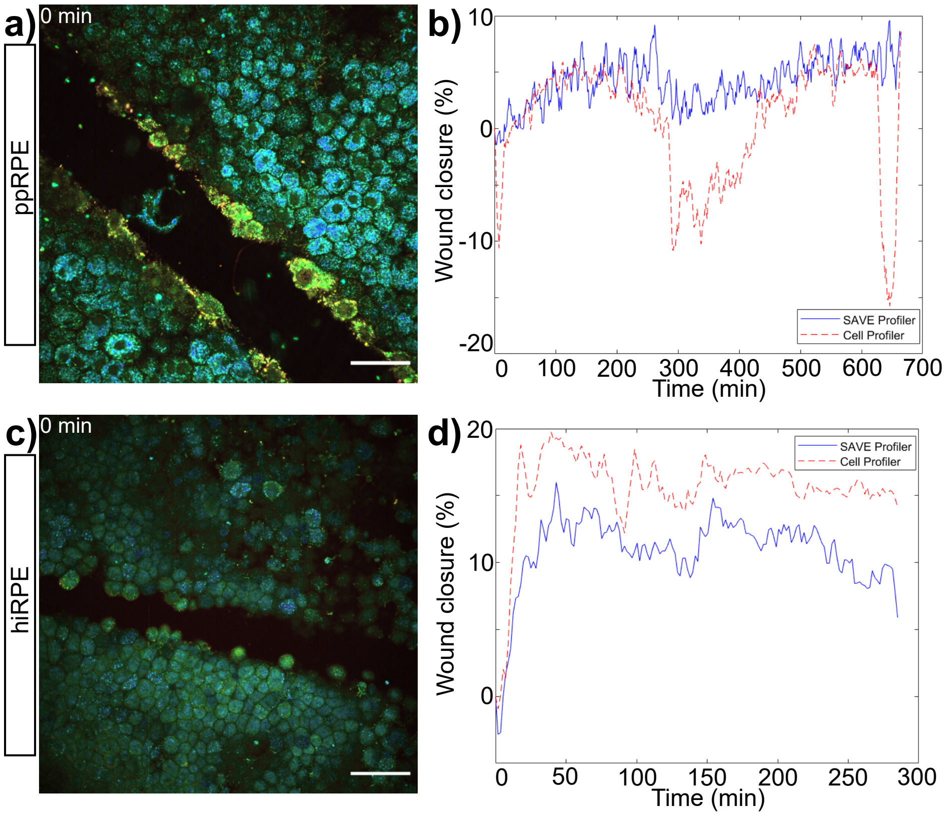

Supplementary Figure 3: **Results of Cell Profiler compared to SAVE Profiler on the samples that failed to close of Fig.2 a)** Beginning of the imaging of a scratch assay failing to close on ppRPE cell culture. **b)** Wound closure calculated with both SAVE Profiler and the Cell Profiler software on the ppRPE sample. **c)** Beginning of the imaging of a scratch assay failing to close on hiRPE cell culture. **d)** Wound closure calculated with both SAVE Profiler and the Cell Profiler software on the hiRPE sample. Note the overestimation of wound closure from Cell Profiler. (Scalebar: 50 μm)

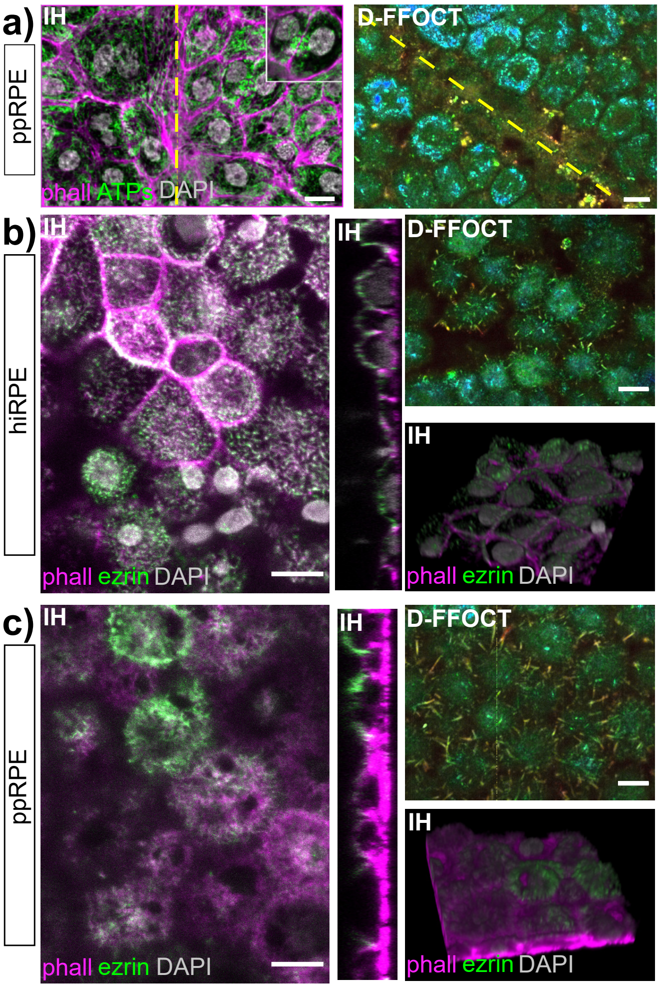

Supplementary Figure 4: **Actin filament behaviours in D-FFOCT and IH. a)** Imaging of a closed scratch assay (yellow dashed line) on a ppRPE sample. Actin filaments labelled with phalloidine (magenta) and mitochondria with ATPs (green) in IH. Corresponding D-FFOCT imaging. **b) & c)** Labelling of microvilli. **b) & c)** Comparison between IH labelling of actin filaments (cytoskeleton and microvilli) with D-FFOCT image. Ezrin is an antibody that uniquely labels microvilli (appearing in green on IH images), while Phalloidin labels actin filaments in general (appearing in magenta on IH images). The IH images clearly show that microvilli are the only structure on top of the cells. **b)** Comparison on a hiRPE sample. From left to right: *en face* IH image showing the distribution of Ezrin and Phalloidin ; cross-section of IH images; D-FFOCT image showing microvilli on top of the cells; 3D rendering of the IH images. **c)** Comparison on a ppRPE sample. From left to right: *en face* IH image showing the distribution of Ezrin and Phalloidin; cross-section of IH images; D-FFOCT image showing microvilli on top of the cells; 3D rendering of the IH images. IH: immunohistochemistry (Scalebar: 10 μm)

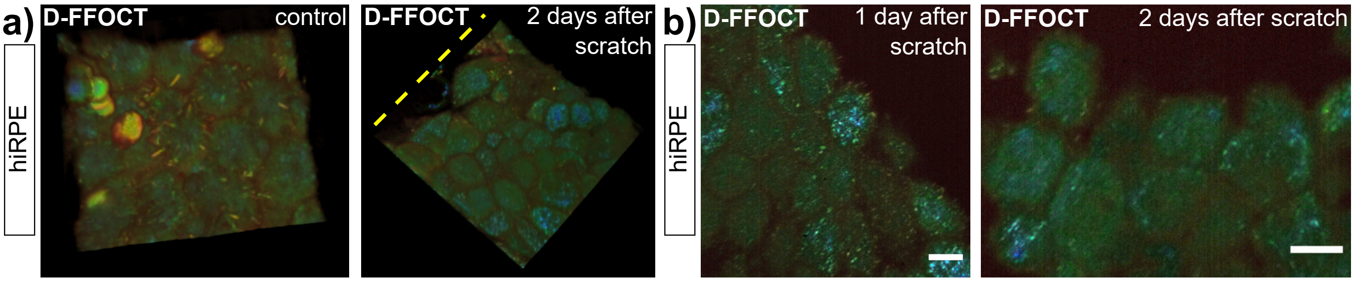

Supplementary Figure 5: **D-FFOCT images 1 or 2 days after scratch assays. a)** Loss of apical processes after scratch assays on hiRPE samples: left, 3D rendering of D-FFOCT images on a control sample exhibiting apical processes (microvilli appear as yellow/red filaments) ; right, 3D rendering of D-FFOCT images on a sample 2 days after the scratch assay, showing no apical processes. **b)** Two different hiRPE samples imaged 1 day and 2 days after scratch, respectively. Both images no longer contain bright cells on the border of the wound. All the cells exhibit a dynamic profile similar similar to unstressed cells. (Scalebar: 10 μm)

Supplementary Table 1: Statistics of the scratch assays for D-FFOCT imaging.

	$\leq 25\mu m$ of (Closing)	$25\mu m \leq . < 100\mu m$ (Failing to close)	$> 100\mu m$ (Expanding)
Total number of samples	6	7	1
Total hiRPE	4	4	1
Wound closure	60 - 70 %	5 - 15 %	< -20 %
Speed	$\simeq$ 8 $\mu m/h$	$\simeq$ 8 $\mu m/h$ (attempt to close)	$\geq$ 30 $\mu m/h$
Total ppRPE	2	3	0
Wound closure	80 - 90 %	5 - 15 %	/
Speed	$\simeq$ 15 $\mu m/h$	$\simeq$ 15 $\mu m/h$ (attempt to close)	/

Supplementary Table 2: Features of the different sample holders

Features / Sample holder	Petri dish: polystyrene	PTFE membranes	PC membranes
Refractive index	1.59	1.31	1.58
Porosity	✗	✓	✓
Adhesion of cells	✓✓	✗	✓
No fringe artefacts	✗	✓✓	✓
Scratch resistance	✓✓	✗	✓

Supplementary movies (available at `https://zenodo.org/record/5894962` [1])

Supplementary Movie 1 : closing ppRPE Closing scratch assay of ppRPE presented in Fig.1.

Supplementary Movie 2 : closing hiRPE Closing scratch assay of hiRPE presented in Fig.1.

Supplementary Movie 3 : closing failure ppRPE Expanding scratch assay of ppRPE, where the cell layer first tends towards wound closure but then finally retracts.

Supplementary Movie 4 : closing failure hiRPE Expanding scratch assay of hiRPE, where the cell layer first tends towards wound closure but then finally retracts.

Supplementary Movie 5 : expanding hiRPE Expanding scratch assay of hiRPE presented in Supplementary Figure 1.

Supplementary Movie 6 : microvilli in real-time, recorded on ppRPE with Holovibes software [2]. Each image is separated from the next by $5ms$. The movements of microvilli are clearly visible.

Supplementary Movie 7 : stacks ppRPE and hiRPE Depth stacks in both ppRPE and hiRPE cell cultures. Differences in the dynamic profile are clearly visible, depending on the maturation stage and origin of the samples. hiRPE (less mature than ppRPE) appear with less pigments, showing a quite homogeneous green dynamic profile inside the cells, while ppRPE appear with blue granules. We can also observe that at the apical surface of the RPE cells (top), microvilli are more present (number, length) and dynamic (i.e. appearing red) on ppRPE than hiRPE.

Supplementary references

[1] GROUX, K. *et al.* Supplementary movies (2022). URL `https://zenodo.org/record/5894962`.

[2] Atlan, M. Holovibes: hologram rendering made easy (2014). URL `http://holovibes.com/`. Http://holovibes.com/.
