# Supplementary material for: Dynamic full-field optical coherence tomography allows live imaging of retinal pigment epithelium stress model
Source: Commun Biol. 2022 Jun 10;5:575. doi: 10.1038/s42003-022-03479-6 (PMC9187748; doi:10.1038/s42003-022-03479-6)
Supplement: Supplementary file 2 — Reporting Summary [file 42003_2022_3479_MOESM2_ESM.pdf]

nature portfolio

Corresponding author(s): Kassandra Groux

Last updated by author(s): Mar 27, 2022

Reporting Summary

Nature Portfolio wishes to improve the reproducibility of the work that we publish. This form provides structure for consistency and transparency in reporting. For further information on Nature Portfolio policies, see our Editorial Policies and the Editorial Policy Checklist.

Statistics

For all statistical analyses, confirm that the following items are present in the figure legend, table legend, main text, or Methods section.

n/a	Confirmed	
☐	☒	The exact sample size (n) for each experimental group/condition, given as a discrete number and unit of measurement
☒	☐	A statement on whether measurements were taken from distinct samples or whether the same sample was measured repeatedly
☒	☐	The statistical test(s) used AND whether they are one- or two-sided<br>*Only common tests should be described solely by name; describe more complex techniques in the Methods section.*
☒	☐	A description of all covariates tested
☒	☐	A description of any assumptions or corrections, such as tests of normality and adjustment for multiple comparisons
☒	☐	A full description of the statistical parameters including central tendency (e.g. means) or other basic estimates (e.g. regression coefficient) AND variation (e.g. standard deviation) or associated estimates of uncertainty (e.g. confidence intervals)
☒	☐	For null hypothesis testing, the test statistic (e.g. F, t, r) with confidence intervals, effect sizes, degrees of freedom and P value noted<br>*Give P values as exact values whenever suitable.*
☒	☐	For Bayesian analysis, information on the choice of priors and Markov chain Monte Carlo settings
☒	☐	For hierarchical and complex designs, identification of the appropriate level for tests and full reporting of outcomes
☒	☐	Estimates of effect sizes (e.g. Cohen's d, Pearson's r), indicating how they were calculated

Our web collection on statistics for biologists contains articles on many of the points above.

Software and code

Policy information about availability of computer code

Data collection	Olympus FLUOVIEW (confocal microscope software)<br>Custom FFOCT software https://zenodo.org/record/3137246 (recording of FFOCT and D-FFOCT images)
Data analysis	Fiji ImageJ1.53c, Imaris x64 software (version 8.4.1 and 9.2.1 Bitplane) for immunohistochemistry images<br>Fiji ImageJ1.53c, custom D-FFOCT analysis codes developped in Matlab (evaluation of different scratch assays parameters, rendering of D-FFOCT images) (https://zenodo.org/record/5820201) for D-FFOCT images

For manuscripts utilizing custom algorithms or software that are central to the research but not yet described in published literature, software must be made available to editors and reviewers. We strongly encourage code deposition in a community repository (e.g. GitHub). See the Nature Portfolio guidelines for submitting code & software for further information.

Data

Policy information about availability of data

All manuscripts must include a data availability statement. This statement should provide the following information, where applicable:

- Accession codes, unique identifiers, or web links for publicly available datasets
- A description of any restrictions on data availability
- For clinical datasets or third party data, please ensure that the statement adheres to our policy

No datasets were used in this study. All data shown in this study are available upon request with Kate Grieve (kategrieve@gmail.com). Videos are available online https://zenodo.org/record/5894962.

Field-specific reporting

Please select the one below that is the best fit for your research. If you are not sure, read the appropriate sections before making your selection.

☒ Life sciences ☐ Behavioural & social sciences ☐ Ecological, evolutionary & environmental sciences

For a reference copy of the document with all sections, see nature.com/documents/nr-reporting-summary-flat.pdf

Life sciences study design

All studies must disclose on these points even when the disclosure is negative.

Sample size	The sample size was determined by the sample holder adapted to our FFOCT technique and to immunohistochemistry experiments (to ease replication).
Data exclusions	No data were excluded from the analyses.
Replication	The experiments done with FFOCT were performed multiple times: the number of samples studied are given in Supplementary information. The immunohistochemistry experiments were performed with at least 3 samples for each set of analysis.
Randomization	Randomization was not relevant to our study: no experimental groups were defined for this study.
Blinding	Blinding was not relevant to our study: the images obtained with FFOCT were compared to immunohistochemistry images in order to determine the origin of the signal observed in our technique's images.

Reporting for specific materials, systems and methods

We require information from authors about some types of materials, experimental systems and methods used in many studies. Here, indicate whether each material, system or method listed is relevant to your study. If you are not sure if a list item applies to your research, read the appropriate section before selecting a response.

Materials & experimental systems

n/a	Involved in the study	
☐	☒	Antibodies
☐	☒	Eukaryotic cell lines
☒	☐	Palaeontology and archaeology
☐	☒	Animals and other organisms
☒	☐	Human research participants
☒	☐	Clinical data
☒	☐	Dual use research of concern

Methods

n/a	Involved in the study	
☒	☐	ChIP-seq
☒	☐	Flow cytometry
☒	☐	MRI-based neuroimaging

Antibodies

Antibodies used

ATPs: ATP Synthase beta Monoclonal Antibody (3D5AB1), Mouse monoclonal, Cat# A-21351, RRID:AB_221512, Thermo Fisher Scientific;

CATSD: cathepsin D (C-20) antibody, Goat polyclonal, Cat# sc-6486, RRID:AB_637896, Santa Cruz Biotechnology;

coxIV: Anti-COX IV antibody [20E8C12], mouse monoclonal, Cat# ab14744, RRID:AB_301443, Abcam;

TGN46 : TGN46 antibody, mouse monoclonal, Cat# ab50595, RRID:AB_2203289, Abcam;

LAMP1 : LAMP1 antibody, rabbit polyclonal, Cat# ab24170, RRID:AB_775978, Abcam;

anti Mouse Donkey polyclonal 488, Cat# A-21202, Invitrogen, RRID AB_141607;

anti Rabbit Donkey polyclonal 594, Cat# A-21207, Invitrogen, RRID:AB_141637;

anti Rabbit Donkey polyclonal 647, Cat# A-31573, Invitrogen, RRID:AB_2536183

anti-Goat Donkey polyclonal Alexa 594, Cat# A-11058, Thermo Fisher Scientific, RRID:AB_2534105

Monoclonal Anti-Ezrin antibody produced in mouse, Sigma-Aldrich Cat# E8897, RRID:AB_476955

Validation

Primary antibodies validations: seller recommendations and relevant citations.

Validation	AB_221512 : seller recommendations: Applications: IF, ICC, WB.<br><br>PMID:16439467, PMID:16730322, PMID:17099894, PMID:17131355, PMID:18344451, PMID:18718527, PMID:18790061, PMID:19481066, PMID:19767061, PMID:19806589, PMID:20074638, PMID:20410436, PMID:20875793, PMID:20940630, PMID:21056597, PMID:21345788, PMID:21531334, PMID:21795630, PMID:21878618, PMID:21895890, PMID:21991365, PMID:22493494, PMID:22504705, PMID:22615909, PMID:23333404, PMID:23453926, PMID:23889209, PMID:24330338, PMID:24874806, PMID:25085991, PMID:25159328, PMID:25168043, PMID:25445031, PMID:25865307, PMID:26031781, PMID:26217791, PMID:26297831, PMID:28712724, PMID:30100261, PMID:30304679, PMID:30362197, PMID:30625316<br><br>AB_637896, seller recommendations: ELISA; Immunohistochemistry; Immunoprecipitation; Immunofluorescence; Immunocytochemistry; Western Blot; WB, IP, IF, IHC(P).<br><br>PMID:25811319, PMID:25855184, PMID:28828399, PMID:30748001, PMID:31006538<br><br>AB_301443, seller recommendations provided: Flow Cyt, IHC-FoFr, WB, ICC/IF, IHC-Fr, IHC-P, IP<br><br>PMID:24914935, PMID:28902411, PMID:29107503, PMID:30293865, PMID:30428348, PMID:31242426, PMID:32573439<br><br>AB_476955 : seller recommendations: Applications: microarray, immunoprecipitation, immunocytochemistry; Immunohistochemistry; ; Western Blot;<br><br>PMID:25271146, PMID:28815590, PMID:31883834<br><br>AB_2203289, seller recommendations provided: western blot, immunoprecipitation, immunocytochemistry<br><br>PMID:30057173, PMID:30122350, PMID:30811981, PMID:31348885, PMID:32778221<br><br>AB_775978, seller recommendations provided: Immunocytochemistry; Immunofluorescence; Immunohistochemistry; Western Blot;<br><br>PMID:27641956, PMID:28552616, PMID:28919207, PMID:29149599, PMID:29804890, PMID:30269989, PMID:30271583, PMID:30311906, PMID:30404007, PMID:30566862, PMID:30612743, PMID:30910009, PMID:31006538, PMID:31061090, PMID:31261758, PMID:31453805, PMID:31848386, PMID:31934854, PMID:32320289, PMID:32671315

Eukaryotic cell lines

Policy information about cell lines

Cell line source(s)	Human induced pluripotent stem cell (hiPSC) derived-RPE cell were generated at the Vision Institute (Paris, France) using established protocol using AHF1pi2 hiPSC clone as described in Reichman et al., 2017 PMID: 28220575.
Authentication	In accordance with French legislation, we certify that the elements or products of the human body or their derivatives obtained have been sampled and/or collected with the prior consent of the donor and that no payment of any form has been provided to the donor for this sample. Handling of donor tissues adhered to the tenets of the Declaration of Helsinki of 1975 and its 1983 revision in protecting donor confidentiality.
Mycoplasma contamination	We confirm that all cell lines were tested negative for mycoplasma contamination.
Commonly misidentified lines (See ICLAC register)	No misidentified cell lines were used in this study.

Animals and other organisms

Policy information about studies involving animals; ARRIVE guidelines recommended for reporting animal research

Laboratory animals	The study did not involved laboratory animals.
Wild animals	The study did not involved wild animals.
Field-collected samples	The study did not involved samples collected from the field.
Ethics oversight	Porcine eyes were bought at a local slaughterhouse (Guy Harang, Houdan, France) in agreement with the local regulatory department and the slaughterhouse veterinarians (agreement FR75105131). This procedure adheres to the European initiative for restricting animal experimentation as not a single animal was killed for our experimentation. Eyes were taken from animals sacrificed daily for human consumption.

Note that full information on the approval of the study protocol must also be provided in the manuscript.
